# A Statistical Approach to Provide Individualized Privacy for Surveys

**DOI:** 10.1371/journal.pone.0147314

**Published:** 2016-01-29

**Authors:** Fernando Esponda, Kael Huerta, Victor M. Guerrero

**Affiliations:** 1 Computer Science Department, Instituto Tecnológico Autónomo de México (ITAM), Mexico City, Mexico; 2 Statistics Department, Instituto Tecnológico Autónomo de México (ITAM), Mexico City, Mexico; Texas A&M University, UNITED STATES

## Abstract

In this paper we propose an instrument for collecting sensitive data that allows for each participant to customize the amount of information that she is comfortable revealing. Current methods adopt a uniform approach where all subjects are afforded the same privacy guarantees; however, privacy is a highly subjective property with intermediate points between total disclosure and non-disclosure: each respondent has a different criterion regarding the sensitivity of a particular topic. The method we propose empowers respondents in this respect while still allowing for the discovery of interesting findings through the application of well-known inferential procedures.

## Introduction

Globalization and the internet era have brought with them a huge array of opportunities for data driven statistical applications and data driven science. An increasing volume of digital information is stored about individuals: their preferences, diseases, relationships, and even their current location; and an even greater amount about phenomena ranging from traffic to radioactivity that is picked up by sensors. Its uses provide great benefits to governments, scientists, health specialists and marketers alike, but by the same token, it has made the preservation of privacy a more urgent matter: data are long-lived, ubiquitously accessible, and with the advent of Big Data mining, exploitable in unimaginable ways. The benefits of the widespread efforts for data collection and its privacy related challenges are well captured by the President’s Council of Advisors on Science and Technology’s report on Big Data and Privacy [1].

Surveys are a useful recourse for collecting data in a directed fashion, be it from individuals or from machines. One important challenge experimenters face is when the data to be collected are sensitive in nature, as the subjects might refuse to participate or could participate with a strong response bias: imagine collecting data related to venereal diseases, or the radioactivity levels in a certain geographical area; or the speed at which your car is being driven. Additionally respondents should be reasonably protected from potentially harmful and unexpected uses of their information.

There are a few recourses to safeguard privacy in such scenarios, among which we have: anonymity, cryptography, and information reduction techniques. Anonymity is a method in which the de-identification of the respondent is guaranteed from the onset; the survey contains all the relevant data except that which can be used to associate the answers to a specific interviewee. The drawbacks of this technique lie in the difficulty of providing such guarantees in an effective and believable manner, respondents might still be weary or even incapable of answering a very sensitive question, and researchers forgo the possibility of conducting longitudinal studies. Anonymity can also be provided after the data collection has taken place (see [2, 3]). The objective of these techniques is to anonymize (de-identify) the sensitive data and allow them to be disclosed; its disadvantage from the vantage point of surveying—during data collection—is that it doesn’t provide many guarantees to entice truthful participation.

Cryptography based methods are generally applied after the survey has been conducted (with the exception of multiparty computation techniques, which we classify in the anonymity group [4]) and their aim is to ensure that the survey data—including the respondent’s information—can’t be examined but by authorized parties. However, authorizations change over time and cryptographic keys can be stolen, misplaced or misused; this, together with the increasing lifetime expectancy of data, makes their long-lived privacy unlikely. From the surveying standpoint cryptographic techniques are hard to explain and therefore to trust by average individuals and, as is the case for anonymity, respondents still have to answer the sensitive question directly (see [5, 6] for examples).

Finally, information reduction techniques are used during the application of the survey and work by requiring less information from the interviewed, enough to compute population statistics but not enough to impute specific sensitive answers to specific respondents. In this way surveys are de-sensitized and respondents can provide their identification data for longitudinal studies without the fear that their answers will come back to haunt them (for examples see [7–14]). Their main disadvantage is that they are not applicable to every kind of survey; that by collecting less information they require bigger samples to maintain accuracy; and that they use a one-size fits all privacy scheme, which squanders information that some respondents may be willing to surrender and forces the more hesitant ones to bias their participation or response. Additionally, techniques such as randomized response techniques and negative surveys have suffered from successfully explaining to respondents how the survey should be answered. However, we believe that this shortcoming is quickly being surpassed by the widespread use of electronic devices that collect data in such a way that the complexity and awkwardness of randomizing devices is hidden from respondents. Furthermore an increasing amount of sensitive data are being collected from sensors to which these techniques can be applied transparently [15–18].

In this paper we focus on an information reduction technique that addresses the fact that the sensitivity of a question or topic is a subjective matter and allows different respondents to disclose a different amount of information for the same question. Our method is a generalization of the Negative Survey technique [10]; we present the surveying technique as well as some of its key statistics and leave the specifics of a survey design outside the scope of this work. We consider that our instrument is appropriate for collecting data from people and from devices, and that it can be applied straightforwardly to the latter but that much work is needed to make its guarantees clear and its administration transparent to the former. Furthermore, we believe that this technique can be successfully employed for answering database queries in a private fashion (where the respondents are the individual fields of each database entry) and thus used for reducing the privacy concerns of already collected data while preserving some of their value. We provide a simulation study using a publicly available database in order to show the accuracy of the technique and how it could be used to collect data—simulating database entries as respondents—or to disclose data sources that contain sensitive attributes.

In Section we briefly explain the Negative Survey technique and follow, in Sections and, with a generalization that allows the experimenter to set the level of privacy to the survey and with a scheme that enables the respondent to decide on its own on the appropriate level. In Section we provide a simplified method for computing the relevant statistics of our instrument for a special design case, which we believe will be widely applicable, and in Section we introduce our instrument that empowers each participant to elect the amount of information to disclose. Section presents the results of our simulation study using real data and we finish with a discussion of the current work and some of its possible directions.

## Negative Surveys

**Fig 1:**
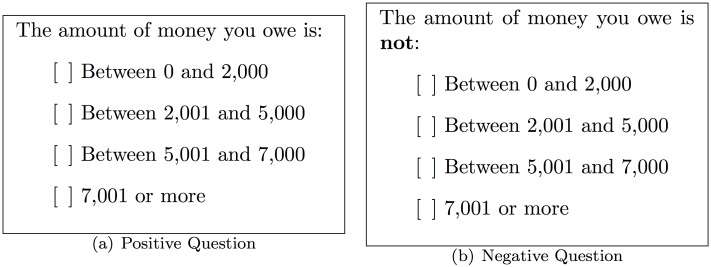
A positive question and its corresponding negatively framed question. Respondents are asked to choose only one option in both cases.

Negative surveys, introduced in [10], is a method for applying a multiple choice questionnaire with *t* exhaustive and mutually exclusive categories—see [19, 20] for refinements on the technique and [21–25] for some applications. The technique is useful when the query in question is sensitive in nature and might cause response and non-response bias. In essence, the approach consists of negating the original question and having respondents choose one among the *t* − 1 options that now apply to him/her with a known probability distribution (see the example in Fig 1). Negative surveys provide a scheme that is expected to help reduce response and non-response biases and that will safeguard sensitive information in the most secure way: by not collecting it in the first place.

Note that not all positive questions can be translated straightforwardly to negative questions as some categories might be sensitive in themselves; for example, when asked “How many sexual partners have you **not** had?” the category “Between 0 and 2” reveals more than some respondents might like. In such scenarios the sensitive category should be replaced with a *dummy* category, a sink so to speak, and the design matrix (see Section) adjusted accordingly.

What is interesting about this method is that even though the negative version provides less information about each respondent, meaningful population statistics can still be estimated. It enables an experimenter to learn something about the population without being able to impute a sensitive answer to a particular individual. However, sensitivity to questions is a relative matter as not everybody places the same burden on the same topic. In the following section we generalize the negative survey scheme in such a way that respondents can decide how much to reveal allowing experimenters to take advantage of the information that is willingly provided.

## Interviewer Defined Privacy: Multiple-answer Questionnaire

In this section we generalize the one-answer negative survey model—where one and only one of the categories must be chosen—to the case in which a respondent chooses *k*_0_ of the available options. We discuss the case in which the corresponding positive setup has *t* exhaustive and mutually exclusive alternatives and where *k*_0_ is previously fixed by the experimenter taking any value between 1, corresponding to the one-answer model, and *t* − 1, corresponding to a positive survey. By letting *k*_0_ vary we have a variety of models for the same question, each affording a different amount of privacy. This scheme may be suitable for disclosing data that were previously collected but for which we wish to provide a certain, uniform level of obfuscation. Later, in Section, we extend this design to allow for a record (or respondent) level of privacy.

As with the one-answer scheme, let *X* be a random variable denoting the category to which the respondent truly belongs—and does not wish to fully disclose. Let *π*_*j*_ = *P*(*X* = *j*) be the probability that *X* takes on the value *j* with *j* ∈ {1, 2, …, *t*}, $\sum_{j=1}^{t}\pi_{j}=1$ and **π** = (*π*_1_, *π*_2_, …, *π*_*t*_)^*T*^.

Let *Y* be a random variable denoting the *k*_0_ categories that the respondent has revealed *not* to belong to. This variable takes its values from the set of all combinations of *t* values taken *k*_0_ at a time. We refer to this space as *Ω*_*k*_0__ with cardinality α=(tk0) and denote each element of *Ω*_*k*_0__ as ${\bar{\omega }}_{i}=(\omega_{i1},\omega_{i2},\ldots ,\omega_{ik_{0}})$. Each *ω*_*ir*_, with *r* = 1, 2, …, *k*_0_, refers to a category that has been discarded by the respondent and ${\bar{\omega }}_{i}$ to the set of all simultaneously discarded categories—the respondent’s answer to the negative survey. Finally exactly one of the events $\left\{Y={\bar{\omega }}_{i}\right\}$ occurs for each application of the negative survey with probability *λ*_*i*_ such that $\sum_{i=1}^{\alpha }\lambda_{i}=1$.

Consider *n* independent repetitions of the experiment and let *N*_*i*_ be the random variable denoting the number of occurrences of $\left\{Y={\bar{\omega }}_{i}\right\}$, then $\sum_{i=1}^{\alpha }N_{i}=n$. Together they constitute the random vector ***N*** which follows a multinomial distribution with parameters *n* and **λ** = (*λ*_1_, *λ*_2_, …, *λ*_*α*_)^*T*^, i.e., ***N*** ∼ Multinomial(*n*,**λ**). We then have

$$\begin{array}{cc} \text{E}\left(N_{i}\right) & =n\lambda_{i}\\ \text{Var}\left(N_{i}\right) & =n\lambda_{i}\left(1-\lambda_{i}\right)\\ \text{Cov}\left(N_{i},N_{j}\right) & =-n\lambda_{i}\lambda_{j}\;\text{if}\;i\neq j. \end{array}$$ (1)

The Maximum Likelihood (ML) estimator for *λ*_*i*_ is given by ${\hat{\lambda }}_{i}=N_{i}/n$ and

$$\begin{array}{ll} \text{E}\left({\hat{\lambda }}_{i}\right) & =\text{E}\left(\frac{N_{i}}{n}\right)=\lambda_{i}\\ \text{Var}\left({\hat{\lambda }}_{i}\right) & =\text{Var}\left(\frac{N_{i}}{n}\right)=\frac{1}{n}\lambda_{i}\left(1-\lambda_{i}\right)\\ \text{Cov}\left({\hat{\lambda }}_{i},{\hat{\lambda }}_{j}\right) & =\text{Cov}\left(\frac{N_{i}}{n},\frac{N_{j}}{n}\right)=-\frac{1}{n}\lambda_{i}\lambda_{j}\;\text{if}\;i\neq j. \end{array}$$ (2)

Assuming each individual answers truthfully we can write the conditional probabilities as

$$p_{ij}=\left\{\begin{array}{cc} 0 & \text{if}\;i=j\\ P(Y={\bar{\omega }}_{i}|X=j) & \text{otherwise} \end{array}\right.$$

and by the Law of Total Probability we can see that the probability of obtaining a specific combination ${\bar{\omega }}_{i}$, of *i* categories for all *i* = 1, 2, …, *t* is

$$\lambda_{i}=\sum_{j=1}^{t}P\left\{Y={\bar{\omega }}_{i}|X=j\right\}\pi_{j}=\sum_{j=1}^{t}p_{ij}\pi_{j}\;\text{for}\;i=1,2,\ldots ,\alpha$$

which we can write in matrix notation as

λ=Pπ, (3)

where ℙ is the design matrix with dimension *t* × *t* whose element (*i*, *j*) is given by *p*_*ij*_.

Notice that for this set up we have α=(tk0) equations and only *t* unknowns and thus the system will be overdetermined for 1 < *k*_0_ < *t* − 1. We therefore use the Moore-Penrose pseudo-inverse to construct our estimator. Let ℙ be the design matrix with known conditional probabilities, $\mathbb{P}=U\Sigma V^{T}$ be its singular value decomposition, and let ${\mathbb{P}}^{†}=V\Sigma^{†}U^{T}$ be the generalized inverse of ℙ with its respective singular value decomposition, so that *U* and *V* are orthonormal matrices, while *Σ* is a diagonal matrix whose elements are the singular (nonnegative) values of ℙ. Then if $\hat{\lambda }={\left(\hat{\lambda_{1}},\hat{\lambda_{2}},\ldots ,\hat{\lambda_{\alpha }}\right)}^{T}$ with the $\hat{\lambda_{i}}s$ estimated by ML, we obtain the following result:

**Proposition 1**. Given the system λ=ℙπ where $\mathbb{P}=U\Sigma V^{T}$, then

$$\hat{\pi }=V\Sigma^{†}U^{T}\hat{\lambda }$$

is the unbiased ML estimator for **π** whose variance is given by

$$\text{Var}\left(\hat{\pi }\right)=\frac{1}{n}V\Sigma^{†}U^{T}\left[\text{Diag}\left(\lambda \right)-\lambda \lambda^{T}\right]{\left(V\Sigma^{†}U^{T}\right)}^{T}.$$

One disadvantage of this method is that the singular value decomposition could be computationally costly when faced with big design matrices. An alternative method for computing the desired estimator can be used when the design matrix ℙ has full rank, then $\left({\mathbb{P}}^{T}\mathbb{P}\right)$ is symmetric positive-semidefinite and we can estimate the population proportions **π** by ML as

$$\hat{\pi }={\left(P^{T}P\right)}^{-1}P^{T}\hat{\lambda }$$

with variance

$$\text{Var}\left(\hat{\pi }\right)=\frac{1}{n}{\left(P^{T}P\right)}^{-1}P^{T}\left[\text{Diag}\left(\lambda \right)-\lambda \lambda^{T}\right]P{\left(P^{T}P\right)}^{-T}$$

using a computationally more efficient method, such as Cholesky decomposition.

### Special Case: Equiprobable Design Matrix

In this section we examine the special case in which each of the *t* − 1 categories from which the respondent can pick is chosen with the same probability to form his/her answer set—for example with the assistance of a randomization device. In this case, assuming individuals answer truthfully and according to instructions, the probability of a respondent choosing a set containing its true category is zero and the probability of selecting a set of size *k*_0_ that does not contain it, is inversely proportional to the number of such subsets

pij=P(Y=ω¯i|X=j)=1t-1k0ifj∉ω¯i0ifj∈ω¯i (4)

where ${\bar{\omega }}_{i}=(\omega_{i1},\omega_{i2},\ldots ,\omega_{ik_{0}})$ denotes the possible subsets from which the individual can choose. We again write the probability for each *λ*_*i*_ as

λ=Pπ.

Let ${\bar{\omega }}_{i}^{\prime }=(\omega_{i1}^{\prime },\omega_{i2}^{\prime },\ldots ,\omega_{it}^{\prime })$ be an indicator row vector of dimension *t*, indicating which categories have been discarded, such that

$$\omega_{ij}^{\prime }=\left\{\begin{array}{cc} 1 & \text{if}\;j\in {\bar{\omega }}_{i}\\ 0 & \text{if}\;j\notin {\bar{\omega }}_{i} \end{array}\right.\;\text{for}\;\text{all}\;j=1,2,\ldots ,t.$$

We can rewrite the design matrix as

P=1t-1k0ω¯1′ω¯2′⋮ω¯α′

which yields a system of *α* equations, with the *i*^*th*^ equation described by

λi=∑j=1tpijπj=1t-1k0ω¯i′π, (5)

with this in mind we can now find a more direct way to estimate **π**.

Let $\lambda_{j}^{\prime }$ with *j* = 1, 2, …, *t* be the set of variables defined by:

$$\lambda_{j}^{\prime }=\sum_{\left\{i|j\in {\bar{\omega }}_{i}\right\}}\lambda_{i}\;\text{for}\;\text{all}\;{\bar{\omega }}_{i}\in \Omega_{k_{0}}$$ (6)

that is, $\lambda_{j}^{\prime }$ is formed by the sum of the proportion of all sets that include category *j* as a member. Substituting in Eq (5) we get a system of *t* equations in *t* unknowns

λj′=1t-1k0∑{i|j∈ω¯i}ω¯i′πforj=1,2,…,t.

Note that for each equation we are adding each *π*_*i*_, with *i* ≠ *j*, a total of (t−2t−k0−1) times and we can thus rewrite the above expression as

λj′=t-2t-k0-1t-1k0∑h≠jπh=k0t-1(1-πj)

Now let *M*_*j*_ with *j* = 1, 2, …, *t* be the number of times some respondents eliminated a set containing category *j*, then $\lambda_{j}^{\prime }=M_{j}/n$ is proportional to the probability of selecting the *j*^*th*^ category. This result is expressed as follows.

**Proposition 2**. Suppose an equiprobable design for a negative survey with *k*_0_ answers, then an unbiased estimator of *π*_*j*_ is given by

$${\hat{\pi }}_{j}=1-\frac{t-1}{k_{0}}{\hat{\lambda }}_{j}^{\prime }\;\text{with}\;{\hat{\lambda }}_{j}^{\prime }=\frac{M_{j}}{n}\;\;\text{for}\;\text{all}\;j=1,2,\ldots ,t$$

with corresponding variance and covariances given by

$$\begin{array}{ll} \text{Var}({\hat{\pi }}_{j}) & =\frac{1}{n}{\left(\frac{t-1}{k_{0}}\right)}^{2}\lambda_{j^{\prime }}(1-\lambda_{j^{\prime }})\\ \text{Cov}({\hat{\pi }}_{i},{\hat{\pi }}_{j}) & \begin{array}{l} =-\frac{1}{n}{\left(\frac{t-1}{k_{0}}\right)}^{2}\sum_{\left\{r|i\in {\bar{\omega }}_{r}\right\}}\sum_{\left\{s|j\in {\bar{\omega }}_{s}\right\}}\lambda_{i^{\prime }}\lambda_{j^{\prime }}\;\text{for}\;i\neq j. \end{array} \end{array}$$

We are particularly interested in obtaining a confidence interval for the population proportion of the sensitive category, say the *j*^*th*^ category. Since *M*_*j*_ is a random variable that behaves as Binomial(*n*, *λ*_*j*′_) for each *j* = 1, …, *t*, we follow the Agresti and Coull’s recommendation (see [26]) of using an adjusted Wald interval whose coverage probabilities are closer to the nominal levels than those of the unadjusted interval. The adjustment amounts to adding two successes and two failures when constructing a 95% confidence interval, but in general consists of replacing ${\tilde{\lambda }}_{j}^{\prime }$ by ${\tilde{\lambda }}_{j}^{\prime }={\tilde{M}}_{j}/\tilde{n}$ with $\tilde{M_{j}}=M_{j}+z_{\alpha /2}^{2}/2$ and $\tilde{n}=n+z_{\alpha /2}^{2}$ where *z*_*α*/2_ denotes the upper *α*/2 percentage point of a unit Normal distribution. Thus we deduce that a 100(1 − *α*)% confidence interval for *π*_*j*_, when *n*, *t* and *k*_0_ are fixed, is given by

$$1-\frac{t-1}{k_{0}}\left[{\tilde{\lambda }}_{j}^{\prime }\pm z_{\alpha /2}^{2}\sqrt{{\tilde{\lambda }}_{j^{\prime }}\left(1-{\tilde{\lambda }}_{j^{\prime }}\right)/\tilde{n}}\right].$$

We conclude this section by analyzing the variance of our estimator in terms of the variance of the corresponding positive survey and the variance added by using a multiple answer negative survey

$$\begin{array}{ll} Var\left({\hat{\pi }}_{j}\right) & =\frac{1}{n}{\left(\frac{t-1}{k_{0}}\right)}^{2}\lambda_{j}^{\prime }\left(1-\lambda_{j}^{\prime }\right)\\ & =\frac{1}{n}{\left(\frac{t-1}{k_{0}}\right)}^{2}\left[\frac{k_{0}\left(1-\pi_{j}\right)}{t-1}\right]\left(1-\left[\frac{k_{0}\left(1-\pi_{j}\right)}{t-1}\right]\right)\\ & =\frac{\pi_{j}\left(1-\pi_{j}\right)}{n}\left(1+\frac{t-k_{0}-1}{\pi_{j}k_{0}}\right). \end{array}$$

In contrast to the one-answer setup, where a good strategy to control the variance is by keeping the number of categories low, for the multiple-answer version we can improve its accuracy by reducing the difference between the number of possible options and the participation parameter *k*_0_, which is to say, the privacy afforded to respondents. Furthermore, this parameter gives greater control to the experimenter as it allows a tradeoff between sample size, number of options, and privacy.

With the multiple-answer model we have a way for the experimenter to control the privacy of a survey. However, privacy is an inherently subjective measure and having *k*_0_ fixed might oblige some to disclose more than what they are comfortable with (risking bias) and others to disclose less and waste potentially useful information. In the next section we build on the present model to address this issue.

## Respondent Defined Privacy: Variable-answer Questionnaire

In the previous sections we examined a setup in which the interviewer established a survey wide level of privacy by specifying the number of options each respondent should choose. In what follows we build on these results to create a design that allows each participant to disclose as much information as he/she is confortable with while still providing useful information to the surveyor.

Let ${\mathbb{P}}_{k}$ be the design matrix for a multiple-answer negative survey scheme where *k* categories, the participation parameter, are eliminated by respondents. Let **π**_*k*_ = (*π*_1*k*_, *π*_2*k*_, …, *π*_*tk*_)^*T*^ be the preference vector for the same scheme, where *π*_*jk*_ refers to the proportion of individuals that prefer category *j*, and let **λ**_*k*_ = (*λ*_1*k*_, *λ*_2*k*_, …, *λ*_*αk*_)^*T*^ be the probability vector of observing each combination of eliminated categories. We now obtain the following result.

**Proposition 3**. Given a personalized negative survey applied to a sample of *n* respondents and a weight vector **ζ** = (*ζ*_1_, *ζ*_2_, …, *ζ*_*t* − 1_)^*T*^, the unbiased ML estimator for the preference population proportion **π** is given by

$$\hat{\pi }=\overset{t-1}{\underset{k=1}{\sum }}\zeta_{k}{\hat{\pi }}_{k}$$

where $\sum_{k=1}^{t-1}\zeta_{k}=1$. Its covariance matrix is given by

$$\text{Var}\left(\hat{\pi }\right)=\sum_{k=1}^{t-1}\frac{\zeta_{k}^{2}}{n}P_{k}\left[\text{Diag}\left(\lambda_{k}\right)-\lambda_{k}\lambda_{k}^{T}\right]P_{k}^{T}.$$

Recall that ${\hat{\pi }}_{k}$ is an unbiased estimator for *k* = 1, …, *t* − 1, therefore

$$\text{bias}\left(\hat{\pi }\right)=\text{E}\left(\sum_{k=1}^{t-1}\zeta_{k}{\hat{\pi }}_{k}-\pi \right)=\pi \sum_{k=1}^{t-1}\zeta_{k}-\pi =0.$$

This models affords great flexibility by allowing the experimenter to weigh each multiple-answer estimator according to different scenarios; for instance:

- Each multi-answer estimator is weighed equally
- Estimators with higher variability, e.g. lower participation parameter, receive lower weight
- The weight is proportional to the number of samples in each multi-answer estimator

and choosing the one with the least variability. The only restriction being that the sum of all weights sum to 1. Once the survey has been conducted the desired proportions $\hat{\pi }$ can be computed as per the methods described in Section.

## A Simulated Survey

In this section we simulate a survey to demonstrate the use and results of our method. As discussed in the previous sections, negative surveys may be used to collect sensitive information from a group of people or a group of sensors; in this example we simulate such a collection process by taking a real database, where each record contains the response of an individual to a survey and apply to it a negative questionnaire, in such a way that the actual value for each record is substituted by a set of “negative” categories— categories that do *not* contain the record’s actual value. We then use these to estimate the frequency of each category for the database and compare it to the actual distribution.

The following simulations use data from the Graphic, Visualization & Usability Center’s (GVU) 8th WWW User Survey, available on-line from [27] which archives the survey responses of 10,108 web users with regards to their general demographic information. Some of the categories are intrinsically sensitive in nature, such as sexual preference and race, and we use them to showcase our technique. We use the variable response setup described in Section and assume a uniform distribution between 1 and *t* − 1 for the privacy preferences —1 being the most private eliminating only 1 category from the possible answers and *t* − 1 the least private which exposes the true, positive category. Each multiple answer estimator is weighed in proportion to the number of responses with a given privacy preference. The code used to generate the simulation is available from the authors upon request. Each simulation was run 100 times with different random numbers and its summary statistics are reported by means of graphs.

**Fig 2:**
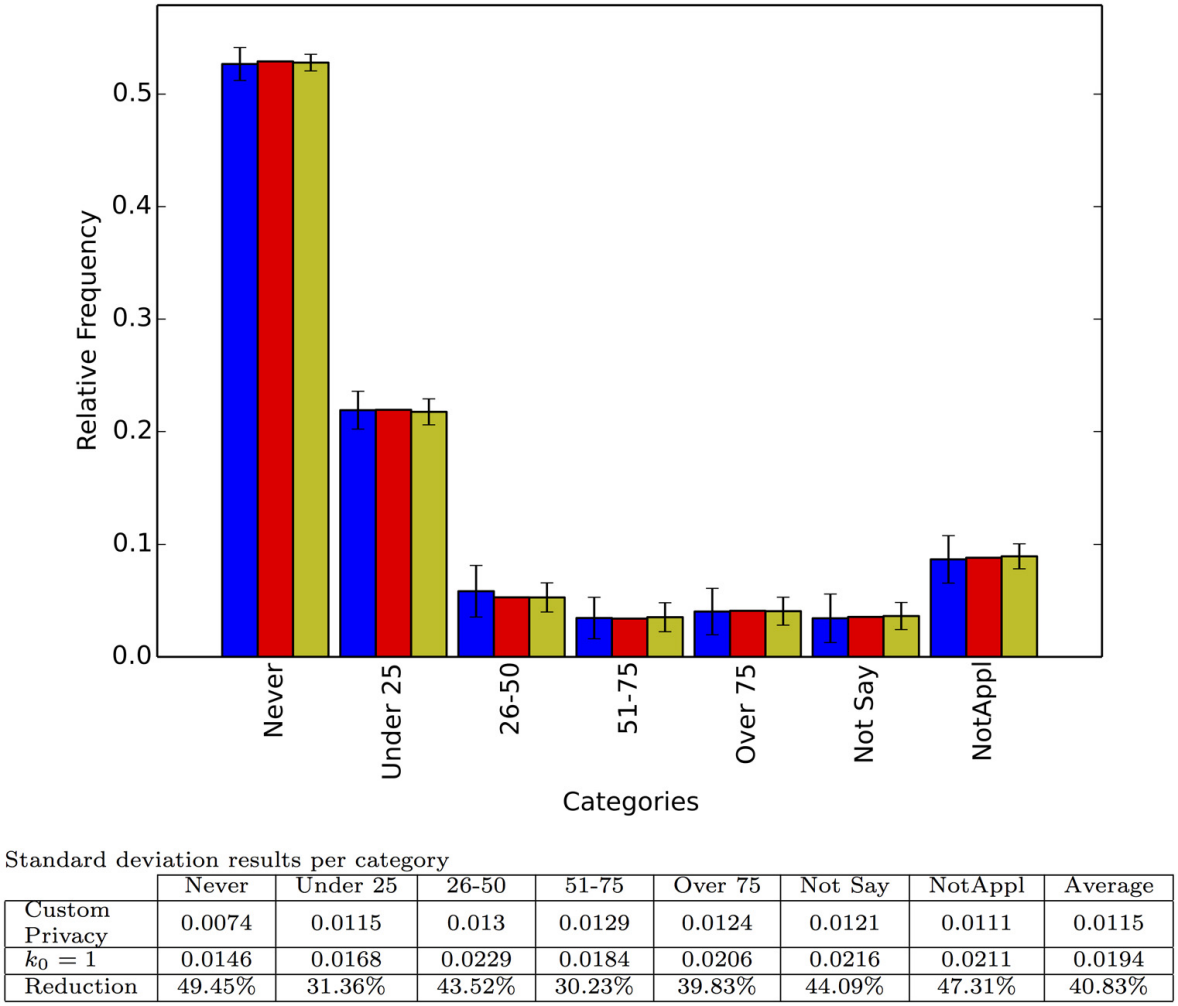
Responses to the question: Some Web sites ask for you to register with the site by providing personal information. When asked for such information, what percent of the time do you falsify the information? Each category shows, from left to right, the relative frequency for the interviewer defined privacy survey with *k*_0_ = 1; the true relative frequency; and the relative frequency for the respondent defined privacy survey with random preference. Each bar represents the average of 100 repetitions and the error bar the standard deviation.

Figs 2 through 6 show the results of the simulations. We selected five different fields from the database corresponding to sensitive questions and whose answers show different distributions, thus allowing us to test our approximation under different scenarios. We also include the results of the interviewer defined privacy survey with *k*_0_ = 1, corresponding to the standard application of a negative survey, to demonstrate how the instrument presented here leverages the extra information disclosed by participants to achieve higher precision (lower variance).

**Fig 3:**
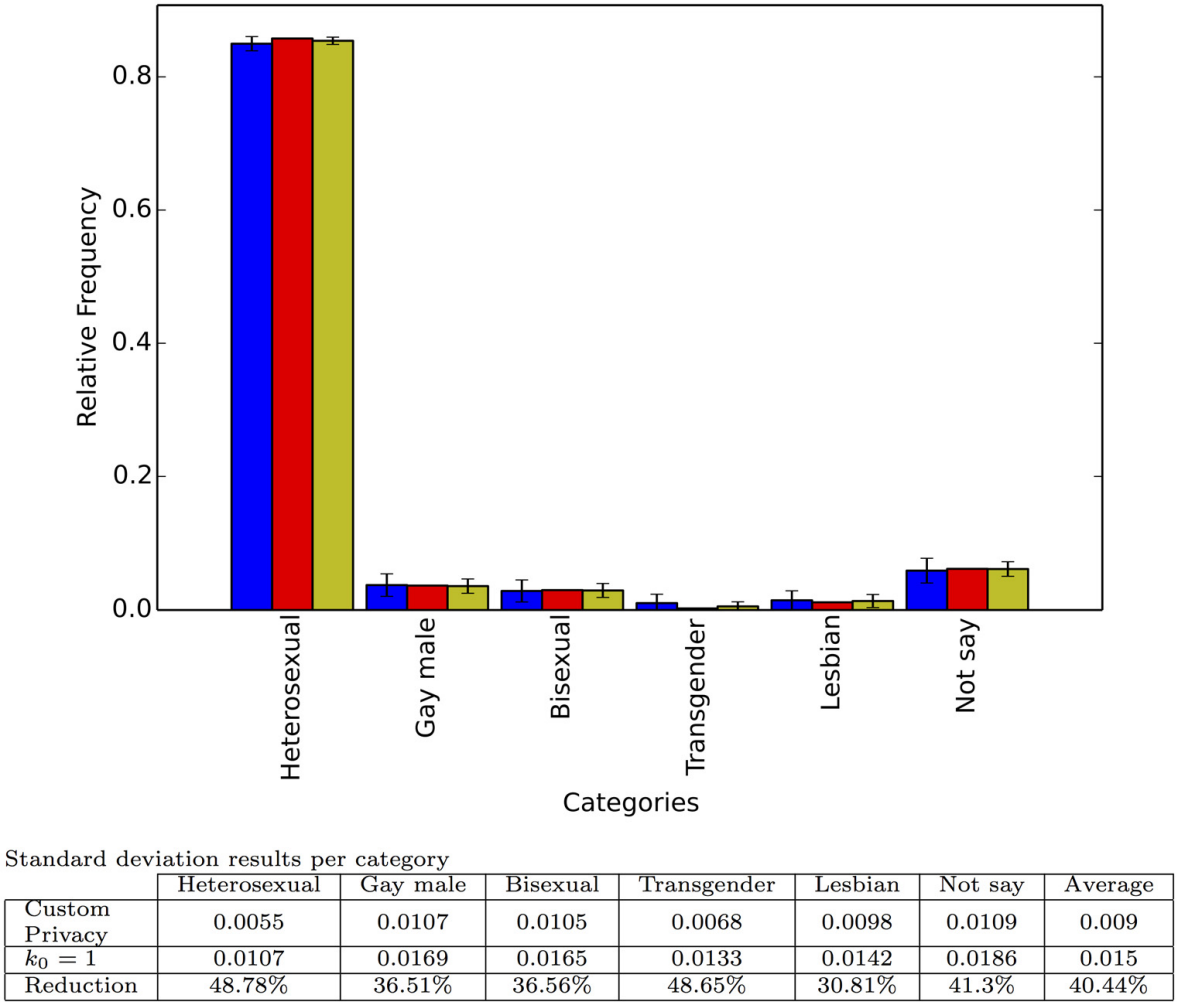
Responses to the question: How would you classify yourself? Each category shows, from left to right, the relative frequency for the interviewer defined privacy survey with *k*_0_ = 1; the true relative frequency; and the relative frequency for the respondent defined privacy survey with random preference. Each bar represents the average of 100 repetitions and the error bar the standard deviation.

**Fig 4:**
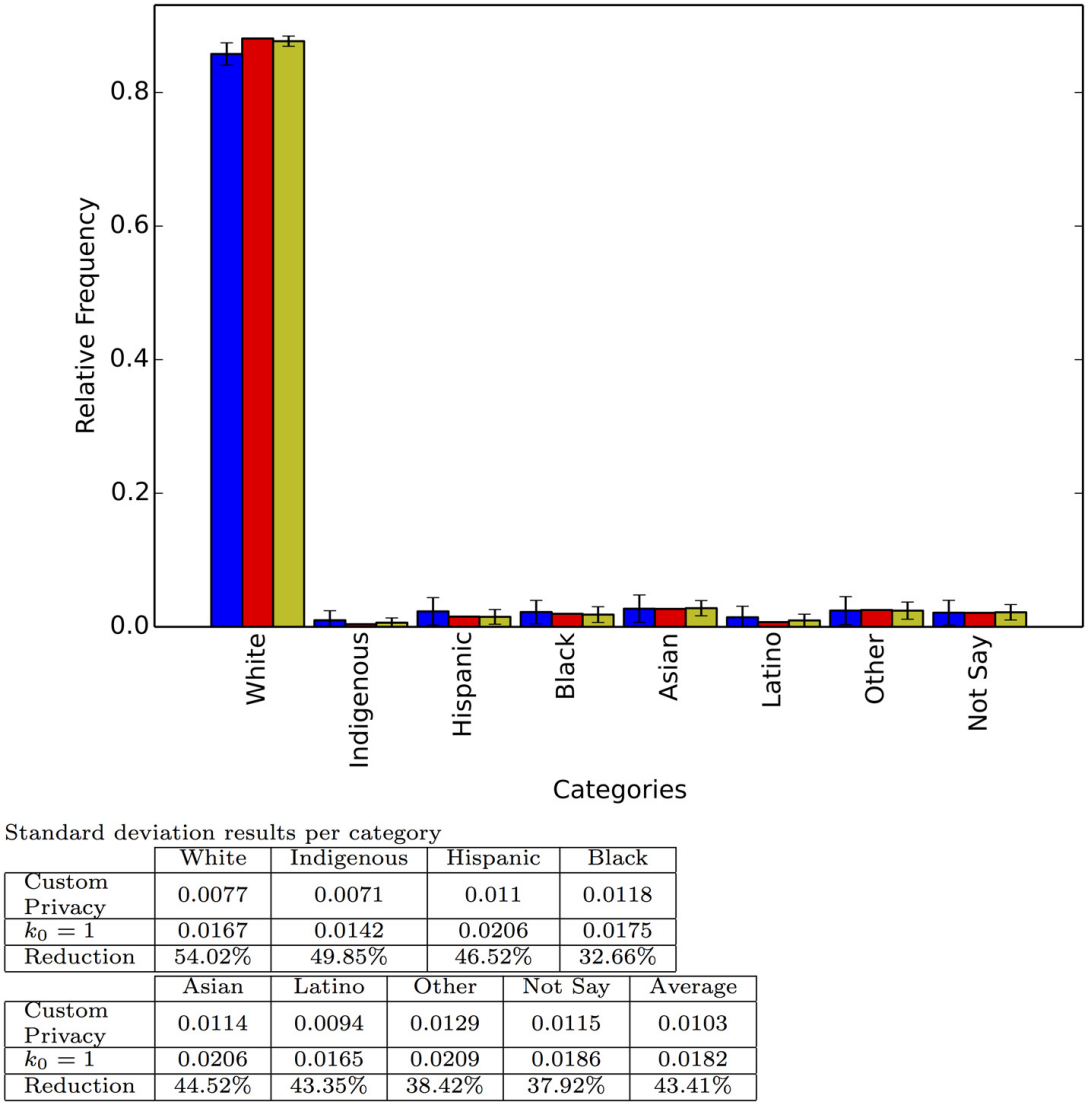
Responses to the question: How would you classify yourself? Each category shows, from left to right, the relative frequency for the interviewer defined privacy survey with *k*_0_ = 1; the true relative frequency; and the relative frequency for the respondent defined privacy survey with random preference. Each bar represents the average of 100 repetitions and the error bar the standard deviation.

**Fig 5:**
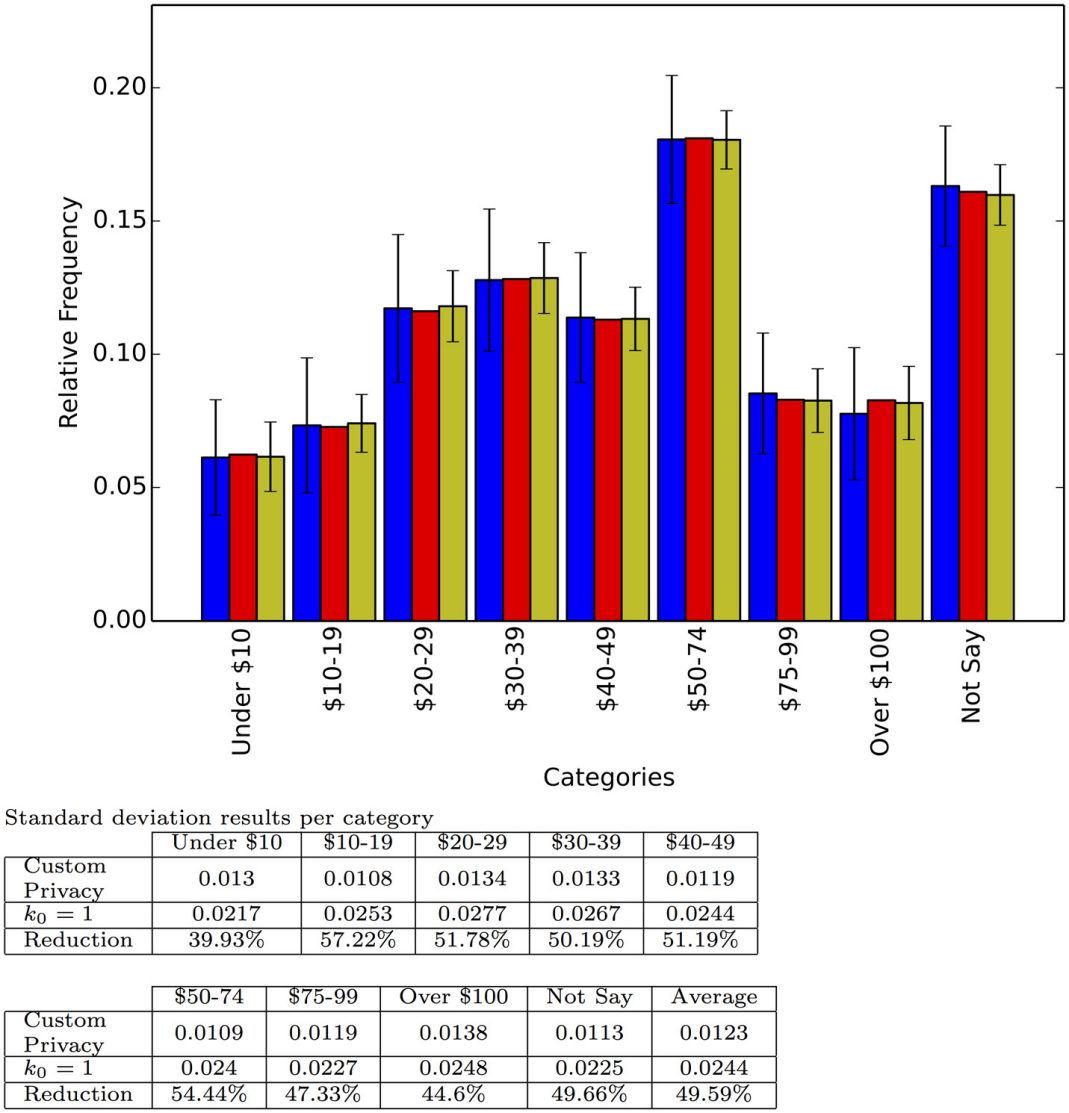
Responses to the question: Please indicate your current household income in U.S. dollars. Each category shows, from left to right, the relative frequency for the interviewer defined privacy survey with *k*_0_ = 1; the true relative frequency; and the relative frequency for the respondent defined privacy survey with random preference. Each bar represents the average of 100 repetitions and the error bar the standard deviation.

**Fig 6:**
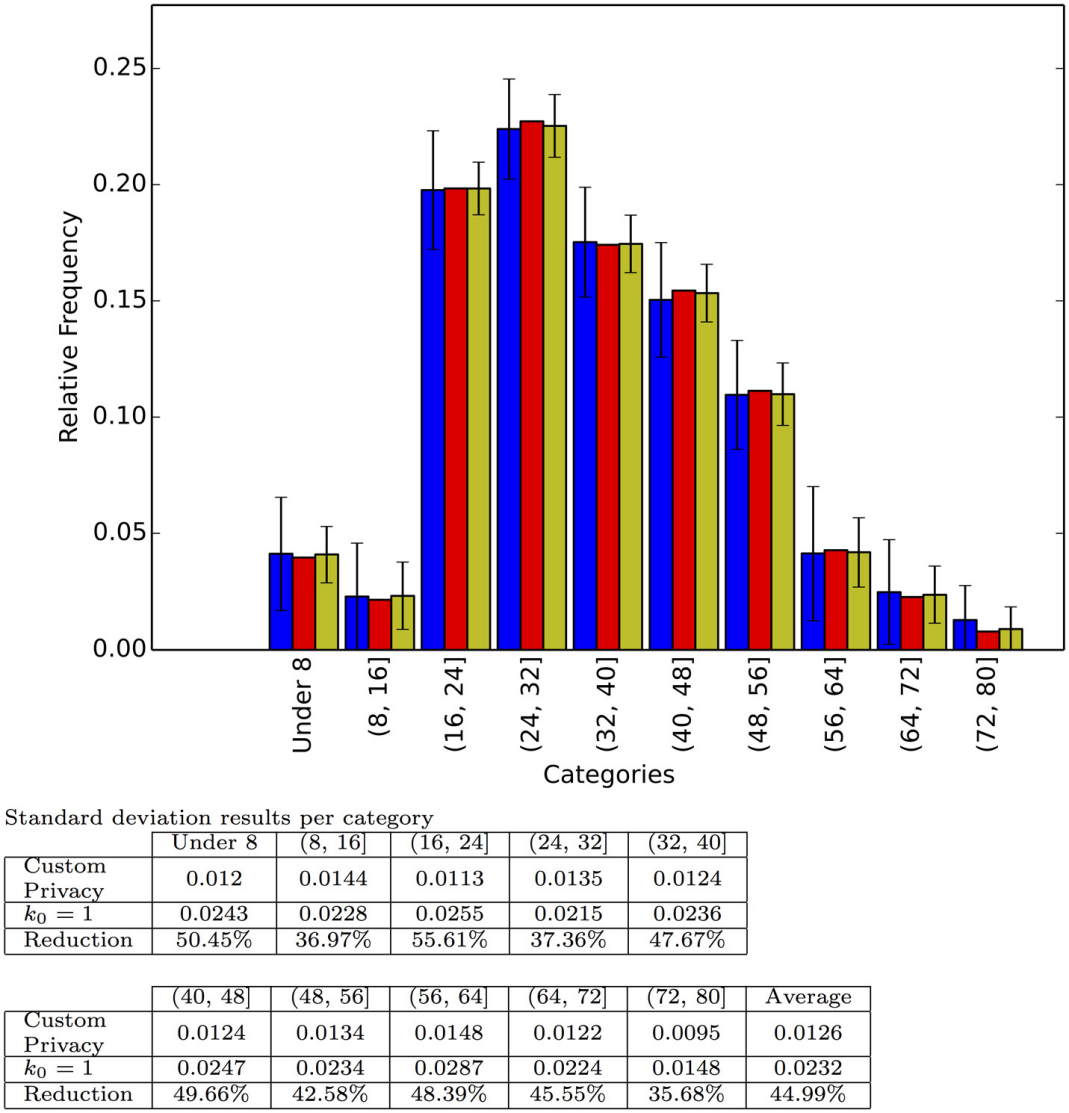
Responses to the question: What is your age? We discretized the answers into 10 equally sized bins and replaced the “Not Say” category for zeros. Each category shows, from left to right, the relative frequency for the interviewer defined privacy survey with *k*_0_ = 1; the true relative frequency; and the relative frequency for the respondent defined privacy survey with random preference. Each bar represents the average of 100 repetitions and the error bar the standard deviation.

**Fig 7:**
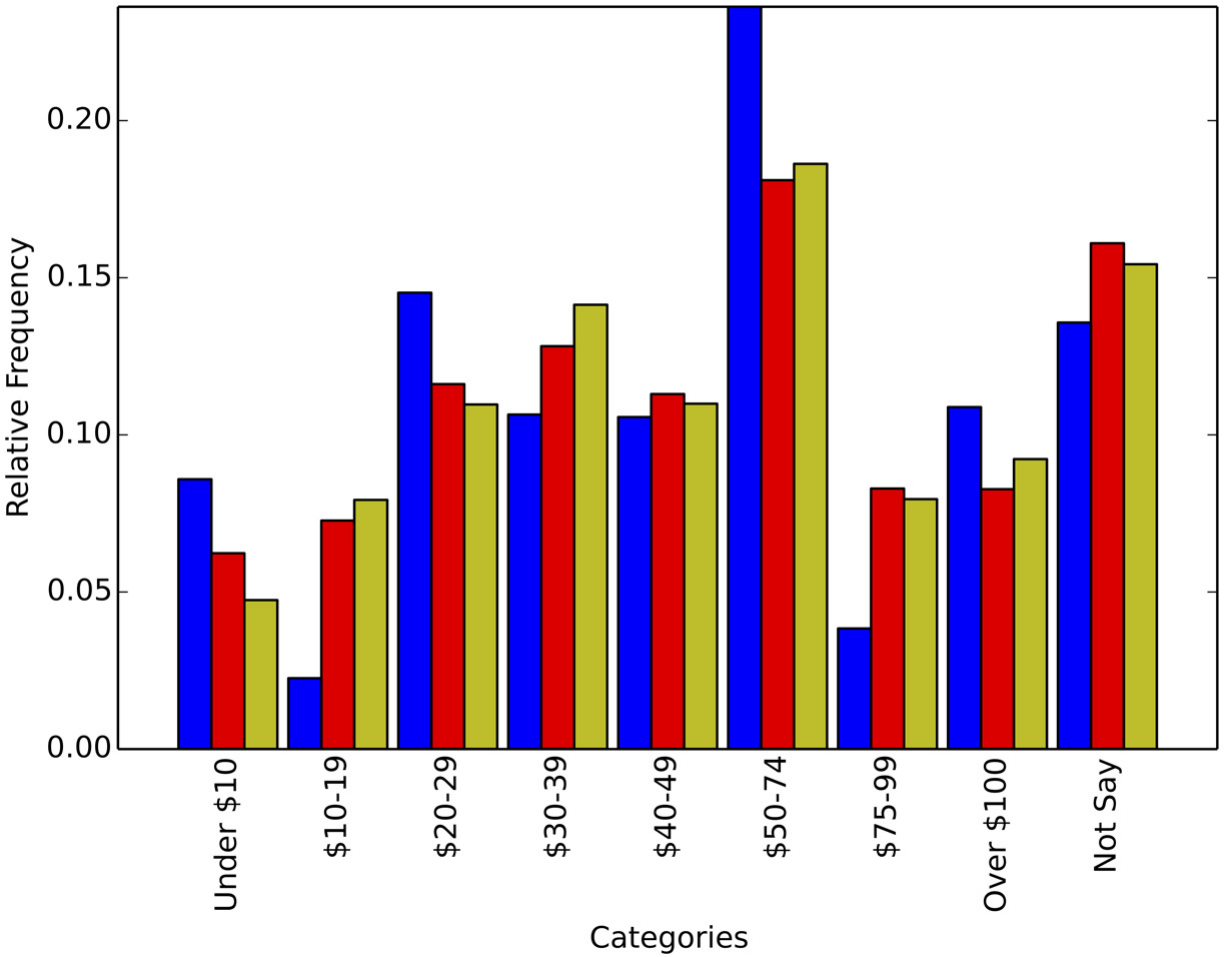
This figure shows the result of only one randomly chosen experiment for the household income question in order to illustrate how the approximation of our method might look for a particular survey. Each category shows, from left to right, the relative frequency for the interviewer defined privacy survey with *k*_0_ = 1; the true relative frequency; and the relative frequency for the respondent defined privacy survey with random preference.

Each experiment was run 100 times and the figures report the average relative frequency estimation as well as the standard deviation of the estimation. As expected, the average estimation is very close to the real relative frequency, but there is marked decrease in the standard deviation (above 40% decrease on average per experiment) from the interviewer defined privacy set up to the respondent defined privacy scheme. This decrease shows how our method is able to harness the extra information provided by some of the participants to reduce its variance. Finally Fig 7 shows the result of one, randomly chosen, simulated survey to illustrate how a particular application of our technique on a particular question might look.

## Discussion

The need for privacy-preserving surveying techniques stems from the desire to eliminate biases related to asking sensitive questions as well as from the duty to protect respondents from unintended consequences. The amount of potentially sensitive data that are now produced daily and stored indefinitely by both humans and sensors has fueled the need for a richer toolkit of data collection techniques. In this paper we introduced a surveying technique that is mindful of the subjectivity inherent in assessing the sensitivity of a question and that empowers a respondent, be it a human or a sensor, to select the amount of information to disclose; in essence, our method allows a question to be answered partially in accordance to its perceived intrusiveness. This technique will enable experimenters to leverage sensitive data in a more efficient way by maintaining sensitivity related biases low without sacrificing the information of those willing to disclose it. Data so gathered will also have a long lasting privacy assurance since, by itself, it is not enough to impute the sensitive characteristic to a particular respondent.

We focused on a specific kind of survey—multiple choice questionnaire with exhaustive and mutually exclusive categories— and based the technique on Negative Surveys. We provided the necessary tools to estimate the population proportion and variance of each category, but left out how the questions on the survey should be worded when the potential respondents are humans rather than electronic devices. Additionally we conducted a simulation study that shows the accuracy of our instrument for real data distributions and points to its possible application for de-sensitizing previously collected sensitive data.
